# Associations Among Diffusion Tensor Image Along the Perivascular Space (DTI-ALPS), Enlarged Perivascular Space (ePVS), and Cognitive Functions in Asymptomatic Patients With Carotid Plaque

**DOI:** 10.3389/fneur.2021.789918

**Published:** 2022-01-04

**Authors:** Hui Liu, Shuai Yang, Wei He, Xiaojuan Liu, Shanyi Sun, Song Wang, Yang Wang, Xiaoliang Zhou, Tao Tang, Jian Xia, Yunhai Liu, Qing Huang

**Affiliations:** ^1^Department of Radiology, Xiangya Hospital, Central South University, Changsha, China; ^2^Hunan Clinical Research Center for Cerebrovascular Disease, Xiangya Hospital, Central South University, Changsha, China; ^3^Department of Emergency, Xiangya Hospital, Central South University, Changsha, China; ^4^Department of Neurology, Stroke Center, Xiangya Hospital, Central South University, Changsha, China; ^5^Department of Integrated Traditional and Western, Xiangya Hospital, Central South University, Changsha, China; ^6^National Clinical Research Center for Geriatric Disorders, Xiangya Hospital, Central South University, Changsha, China

**Keywords:** carotid plaque, carotid atherosclerosis, glymphatic system, DTI-ALPS, ePVS

## Abstract

**Background and Aim:** Carotid atherosclerosis (CAS) is a common pathogenesis of cerebrovascular disease closely related to stroke and silent cerebrovascular disease (SCD), while the insufficient brain perfusion mechanism cannot quite explain the mechanism. The purpose of this study was to utilize diffusion tensor image analysis along the perivascular space (DTI-ALPS) to evaluate the glymphatic system activity and correlated DTI-ALPS with enlarged perivascular spaces (ePVS), carotid intima-media thickening (CIMT), mini-mental state examination (MMSE), and serological indicator in individuals with carotid plaque.

**Methods:** Routine MRI and diffusion tensor images scan of the brain, carotid ultrasound, and blood examination were conducted on 74 individuals (52 carotid plaque subjects, 22 non-carotid plaque subjects), whose demographic and clinical characteristics were also recorded. DTI-ALPS index between patients with carotid plaque and normal controls were acquired and the correlations with other variables were analyzed.

**Results:** The values of ALPS-index in the carotid plaque group was significantly lower compared to normal controls (2.12 ± 0.39, 1.95 ± 0.28, respectively, *p* = 0.034). The ALPS-index was negatively correlated with the basal ganglia (BG)-ePVS score (*r* = −0.242, *p* = 0.038) while there was no significant difference in the centrum semiovale (CSO)-ePVS score. Further analysis showed that there are more high-grade ePVS in the BG compared to the carotid plaque group than in the non-carotid plaque group (84.6% vs. 40.9%, *p* = 0.001).

**Conclusions:** ALPS-index reflects the glymphatic system of the brain, which is associated with early high-risk cerebrovascular diseases. There may be damage in the function of the glymphatic system which induces the expansion of the perivascular space (PVS) in the BG in individuals with carotid plaque.

## Introduction

Carotid atherosclerosis (CAS) can lead to stroke, vascular cognitive impairment, and silent cerebrovascular disease (SCD) (1, 2). CAS is a dynamic evolution which can be divided into three main stages, including carotid intima-media thickening (CIMT), carotid plaque formation, and carotid stenosis. CIMT or carotid plaque formation is a subclinical state of CAS. Studies have shown that the two states may be correlated with cognitive impairment in the high-risk asymptomatic individuals who develop stroke. However, the mechanism has not been fully explained (3, 4). A more recent study has shown that enlarged perivascular space (ePVS) is an early neuroimaging diagnostic biomarker of mild cognitive impairment (5). PVS refers to the normal fluid-filled anatomical structure surrounding the small blood vessels in the brain. Recent studies have shown that PVS is a drainage channel, that plays a critical role not only in the fluid exchange, but also in the removal of waste in the brain and in the maintenance of brain homeostasis and immune regulation. Studies have shown that ePVS increases with age and blood pressure, especially in intracranial atherosclerosis (6, 7). Some cross-sectional studies have also shown that the more ePVS, the more severe white matter hyperintensity (WMH) and lacunar infarction are found in the brain. Furthermore, ePVS are more common in patients with dementia than in the normal people. However, the formation mechanisms of ePVS is still unclear (8, 9).

A recently discovered waste drainage system in the central nervous system, the glymphatic system, has been found to play a significant role in the movement of the cerebrospinal fluid (CSF) along the PVS. This system not only promotes the clearance of accumulated soluble proteins, but also assists in the distribution of nutrients and neuromodulators in the brain. In this glymphatic loop circuit, CSF and interstitial fluid (ISF) interchange by the influx of CSF along the PVS (10–12). The impairment of the glymphatic system has been suggested to cause SCD, cerebral vascular atherosclerosis, neurodegenerative diseases, and other diseases related to aging (13–15).

There is also a growing interest in the potential imaging-marker for glymphatic activity [the diffusion tensor image analysis along the perivascular space (DTI-ALPS) parameter] using diffusion MRI imaging (16, 17). The DTI-ALPS showed promising results in its ability to differentiate glymphatic system impairment in patients with Parkinson's disease, idiopathic normal pressure hydrocephalus, and Type 2 diabetes mellitus (T2DM) (18–20). Previous studies also showed significant positive correlations between DTI-ALPS and cognitive functions assessed with the MMSE in individuals who are cognitively normal and who have Alzheimer's disease (21).

Carotid atherosclerosis, the causative roles of the cerebrovascular dysfunction mechanism, following abnormal vascular endothelial cell damage, lipid deposition, release of inflammatory factors, results in artery stenosis, or even occlusion. Not only stable plaque but also vulnerable plaque cause wide health problems and established risk factors for stroke and vascular cognitive decline in elderly people and in people with vascular Parkinsonism (3, 4, 22).

Previous studies of our group have shown that the cognitive score of patients with carotid artery vulnerable plaque were different from that of the group with stable carotid plaque, while there was no significant difference in the blood pressure and cerebral blood flow perfusion between the two groups (9, 21). By comparing different brain regions in the resting state, the functional MRI (fMRI) between the two groups and local consistency in patients with carotid artery vulnerable plaque were higher than in the stable plaque group, and functional connections in the default network were significantly enhanced. These studies indicated that the corresponding brain regions in the vulnerable plaque group are not in a state of low activity due to insufficient blood supply (6); thus we speculated that cognitive impairment might be induced not only based on hemodynamic changes, but might be due to a new mechanism that causes the changes. We hypothesize that subclinical state of CAS, the carotid plaque, which can be used to assess the vascular risk of asymptomatic individuals and SCD, may induce changes in the glymphatic function of the brain. Therefore, we aimed to evaluate the activities of glymphatic system in patients with DTI, who have carotid plaque, using the DTI-ALPS method. We also investigated whether there were any relationship between the DTI-ALPS-index and ePVS, as well as with MMSE score and the inflammation factors.

To our knowledge, up to now, there is no research to evaluate the function of the glymphatic system in patients with carotid plaque. This study is based on the hypothesis that the state of carotid artery plaque may not change the cerebral perfusion, but the cause of early white matter fiber damage may be caused by the impaired function of the glymphatic system in the group with carotid artery plaque.

## Materials and Methods

### Study Subjects

**Figure 1:**
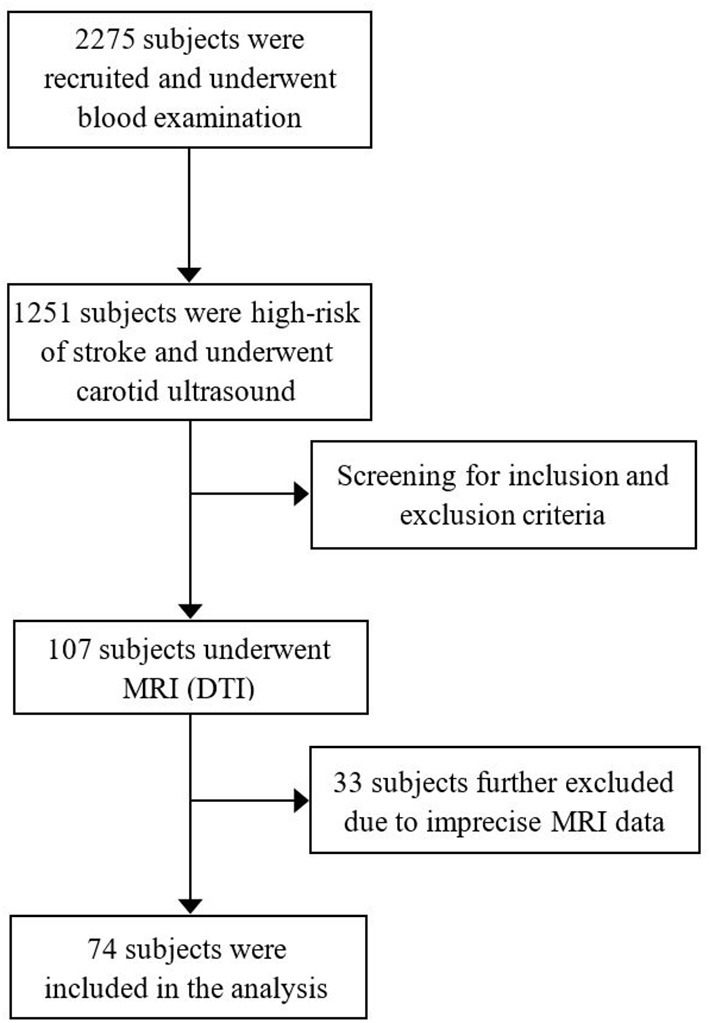
Procedure for checking the enrolled population.

From December 2015 to December 2017, all subjects were recruited from the project of Stroke Risk Screening and Prevention Project in Hunan Province. A total of 2,275 subjects were surveyed to collect the data regarding their gender, age, educational level, smoking status, and previous medical and medication history. Blood samples were also collected. Physical examination and neuropsychological tests (MMSE) were also performed by professional neurologists. All subjects came from the Wujialing community, Changsha city, Hunan province. About 1,251 of the subjects were at high-risk of stroke (participants who were over 40 years old and preserved ≥3 of traditional vascular risk factors were defined as high-risk of stroke) and completed carotid ultrasound examination. According to the inclusion and exclusion criteria, as defined in the following standard, a total of 107 subjects underwent brain multimodal MRI scanning (i.e., T1WI, T2WI, and FLAIR DTI sequences) (Figure 1). The inclusion criteria were as follows: (1) subjects aged from 40 to 75 years old; (2) right handedness; (3) single type of carotid plaque, hypoechoic, isoechoic, or hyperechoic; (4) carotid stenosis <50%; (5) no previous history of stroke or transient ischemic attack; (6) no other organic or mental diseases in the nervous system; (7) no serious heart disease, such as atrial fibrillation; (8) no drug dependence, and (9) education level >6 years. The exclusion criteria were as follows: (1) MRI contraindications; (2) with subclavian artery plaque; (3) have a history of carotid endarterectomy; (4) a history of nervous system trauma; (5) using hormone contraceptive or in pregnancy and lactation; (6) Fazekas scores >2. According to the inclusion and exclusion criteria, 107 subjects were scanned by carotid ultrasound and MRI (i.e., DTI-ALPS). In the end, 74 people (32 men and 42 women; mean of 60.8 years old; age range of 46–76 years) were enrolled in this study. All procedures were approved by the ethics committee of Xiangya Hospital of Central South University. Each participant had given a written informed consent.

### Clinical Information at Baseline

Age, gender, hypertension, diabetes mellitus (DM), hyperlipidemia, body mass index (BMI), coronary heart disease, and smoking status were recorded. Hypertension was defined as the mean systolic blood pressure (SBP) ≥140 mmHg and/or mean diastolic blood pressure (DBP) ≥90 mmHg at two consecutive non-invasive measurements with an interval of at least 15 min, or with a medical history of oral medication with blood pressure lowering drugs. The DM was defined as fasting plasma glucose (FPG) ≥7.9 mmol/L, plasma glucose at any time of ≥11.0 mmol/L, or a history of oral medication of glucose lowering drugs. Hyperlipidemia was defined as low density lipoprotein ≥160 mg/dL, total cholesterol of ≥240 mg/dL, triglycerides of ≥150 mg/dL, or a history of oral medication with lipid-lowering drugs. Coronary heart disease was defined as the presence of clinical manifestations of myocardial infarction, angina, or ischemic heart failure as well as coronary atherosclerosis. Smoking was defined as smoking ≥10 cigarettes per day or smoking for >1 year. Each subject was scored with MMSE by the neurologist trained in professional neuropsychological scoring. MMSE total score was 30 points (23), where the score <27 was considered to be a cognitive impairment.

### Blood Examination

Venous blood was collected from the antecubital vein in the morning before the participants ate breakfast, following an overnight fast of 12–14 h. All subjects underwent routine laboratory tests, including assays for FPG, serum total cholesterol, serum triglycerides (TG), serum low density lipoprotein cholesterol (LDL-c), high-sensitivity C-reactive protein (hsCRP), and interleukin-6 (IL-6).

### Carotid Ultrasound Examination

Carotid ultrasound was carried out with an ultrasound scanner (5-MHz Sector Array transducer; iU22, Philips Ultrasound, Bothell, WA, USA) in both the right and left common carotid arteries (CCAs) and bifurcations by an experienced ultrasound doctor. The distance from the media-adventitia interface to the intima-lumen interface was defined as the intima-media thickness (IMT). A plaque was defined as a focal structure with an IMT >1.5 mm (24). The carotid intima-media thickness (CIMT) was defined as the average IMT and was calculated from the bilateral IMTs of the CCAs, carotid bulb, and bifurcations in a region free of plaque (25).

### MRI Protocol

Magnetic resonance imaging was acquired by using a 3.0-T scanner (SIGNA HDX, GE, Boston, USA) with a 12-channel head coil. The MRI scanning included T1-weighted imaging (T1WI), T2-weighted imaging (T2WI), and fluid attenuated inversion recovery (FLAIR) sequences. Each scan involved a three-dimensional sagittal brain volume imaging (BRAVO) sequence with the following parameters: repetition time (TR) = 7.792 ms, echo time (TE) = 2.984 ms, flip angle = 7°, 188 slices, slice thickness = 1 mm, slice spacing = 1 mm, acquisition matrix = 256 × 256, voxel size = 111 mm^3^. Diffusion tensor imaging (DTI) parameters are as follows: repetition time (TR) = 12,000 ms, echo time (TE) = 72.5 ms, acquisition matrix = 256 × 256, field of view (FOV) = 230 × 230 mm, slice thickness = 3 mm, flip angle = 90, gradient direction = 32, diffusion sensitivity coefficient (b) = 0.1000 s/mm^2^. All the digital imaging and communications in medicine (DICOM) images are imported to RadiAnt DICOM Viewer.

### Enlarged Perivascular Space Scoring

**Figure 2:**
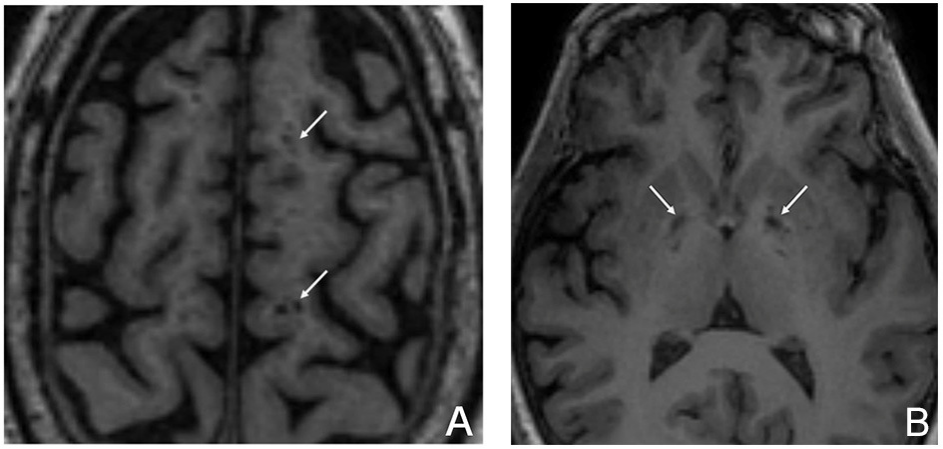
ePVS in the centrum ovale **(A)** and in the basal ganglia **(B)** (arrow).

The layer with the largest number of ePVS was selected independently in the centrum ovale and in the BG. The ePVS was determined according to the following criteria: (1) depending on the image plane, the ePVS was spotty or strip-like, with clear and smooth borders and usually <3 mm in diameter; (2) the ePVS was consistent with the passage of the perforating vessels; and (3) on all MRI sequences, the density of the ePVS was equal to that of the CSF (Figure 2). The ePVS of each region was graded as follows: 0, none; 1, ≤ 10; 2, 11–20; 3, 21–40; 4, ≥40 (26, 27).

### DTI-ALPS Processing and Image Analysis

**Figure 3:**
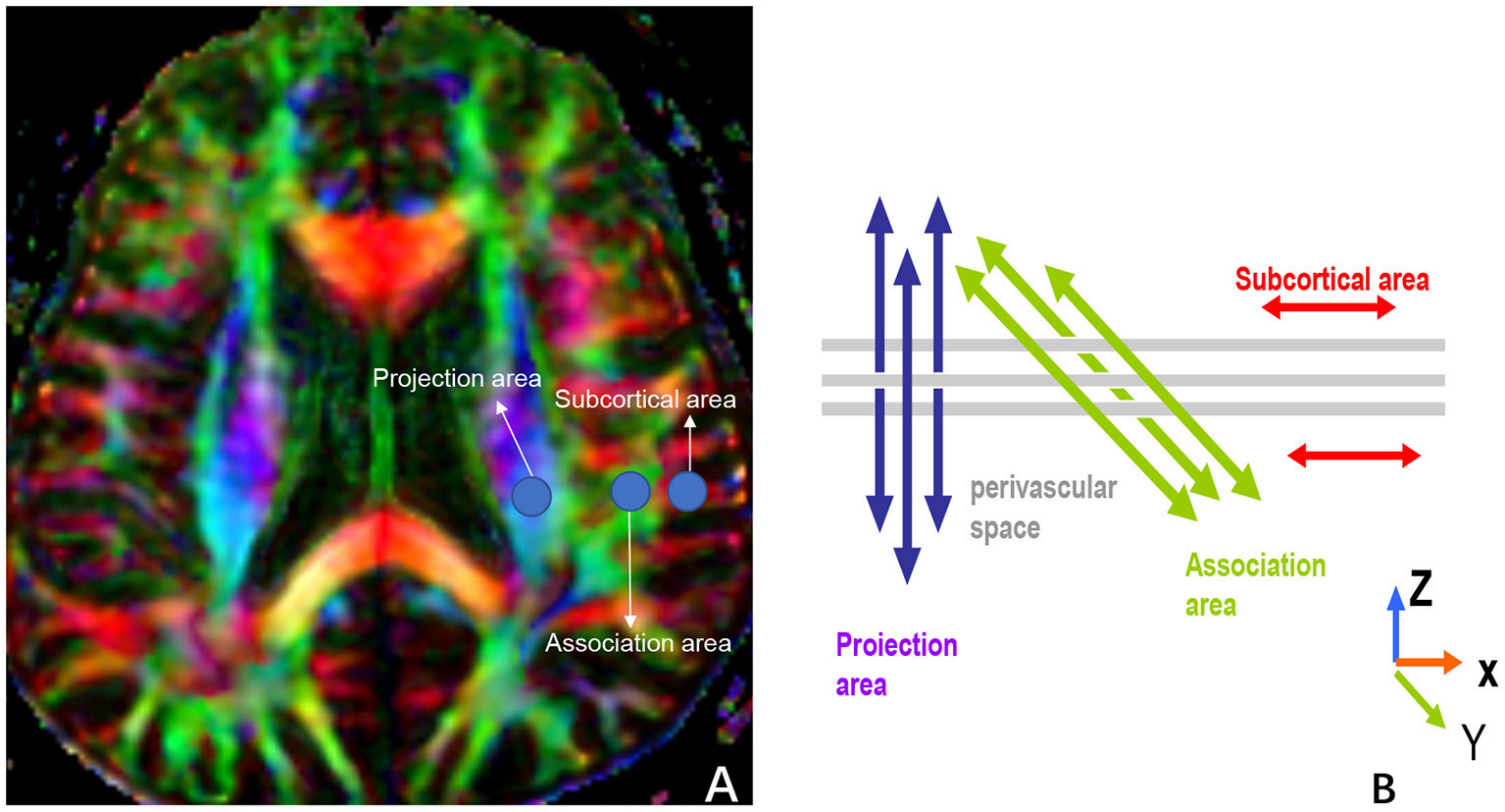
**(A)** shows three ROIs that are placed in the area with projection fibers (projection area), association fibers (association area), and subcortical fibers (subcortical area) to measure diffusivities of the three directions (x, y, z). **(B)** indicates the relationship between the direction of the perivascular space (gray cylinders) and the directions of the fibers [**(B)** uses Figure 2b in the paper, “*Evaluation of glymphatic system activity with the diffusion MR technique: diffusion tensor image analysis along the perivascular space (DTI-ALPS) in Alzheimer's disease cases*” for reference].

The ALPS-index was calculated to assess the activity of the glymphatic system. We adopted the method for DTI-ALPS processing and measurement from the previous publication (7–9, 21, 28, 29). Diffusion metric images were generated by using dTV.II.13k+ software (Department of Biomedical Information Sciences, Graduate School of Information Sciences, Hiroshima City University) (30). The software creates computational images of the diffusion tensor including a color-coded fractional anisotropy (FA) map and diffusivity map, and in addition, has the capability to calculate diffusivity in the direction of the x-, y-, and z-axes on each image. We selected the slice at the level of the lateral ventricle body (Figure 3A). At this level, the direction of the perivascular space is perpendicular to the ventricle wall and is thus mostly in the right-left direction (x-axis) on the axial plane. The direction is also perpendicular to the direction of both the projection fibers (mostly in the z-axis) and the association fibers (mostly in the y-axis). Thus, the diffusivity along the x-axis at regions with projection/association fibers will at least partly represent the diffusivity along the perivascular space. A 5-mm diameter spherical region of interest (ROI) was placed in the area of the projection fibers, the area of the association fibers, and the area of the subcortical fibers in the left hemisphere (Figure 3B). For each area, we calculated the diffusivity in the directions of the x-, y-, and z-axes and the index value was derived from the ratio of the two diffusivity value sets that are perpendicular to the main fibers in the tissue: that is, the ratio of the average values of the x-axis diffusivity in the area of the projection fibers (Dxproj) and the x-axis diffusivity in the area of the association fibers (Dxassoc) to the average value of the y-axis diffusivity in the area of the projection fibers (Dyproj), and the z-axis diffusivity in the areas of the association fibers (Dzassoc), as shown below:


$$\begin{array}{l} ALPS-index=mean(Dxproj,\;Dxassoc)/(mean\;(Dyproj,\;Dzassoc) \end{array}$$

### Repeatability Assessment

The ePVS SCORING and ALPS-index in our project were measured by two radiologists (with 10 and 15 years of experience in neuroimaging, respectively). Intra-class correlation coefficient (ICC) and Bland-Altman plot (mean difference, 95% limits of agreement) were used to assess the agreement between the two radiologists. For ePVS SCORING, The ICC coefficients of inter-class and intra-class were 0.93 and 0.91, respectively. For ALPS-index, The ICC coefficients of inter-class and intra-class were 0.86 and 0.81, respectively. Bland-Altman plot showed that only 1 plot is outside of the 95% conformance limitation for ePVS SCORING and ALPS-index, respectively. At last, the two radiologists compared their ratings and decided on the final data together.

### Statistical Analysis

All the data were analyzed by SPSS 22.0 software, the continuous variables were expressed by mean value ± standard deviation. Shapiro Wilk (S–W) test was used to test the normally distributed variables, independent sample *T*-test was used for two sample variables, Mann–Whitney *U*-test for non-normal distribution variables, and χ^2^ test for type variables. Pearson's correlation analysis was used to analyze the correlation between the two sample variables, and Spearman's correlation was used to analyze the rank variables. The value, *P* < 0.05 means that the difference is statistically significant. An independent samples *t*-test and Wilcoxon rank-sum test were used for evaluating patient characteristics. The statistical significance levels of the report were two-sided, with the statistical significance set at 0.05.

## Results

According to the carotid ultrasound examination, 74 people (32 men and 42 women; mean of 60.8 years old; age range of 66–76 years) were allocated into two groups (non-carotid plaque group and carotid plaque group). Demographic and clinical characteristics of the two groups are shown in Table 1.

**Table 1:** Demographic and clinical characteristics of the patients.

	**Non-carotid plaque group (*n* = 22)**	**Carotid plaque group (*n* = 52)**	**χ^**2**^/*t* value/ *z* value**	***P*-value**
Age (year), mean (SD)	58.77 ± 71.16	61.65 ± 6.79	−1.641	0.105
Male sex no. (%)	11 (50)	11 (21)	0.582	0.445
Current cigarette smoker no. (%)	5 (22.7)	15 (28.8)	0.293	0.588
Hypertension no. (%)	15 (68.2)	32 (61.5)	0.294	0.587
Diabetes no. (%)	7 (31.8)	20 (38.5)	0.294	0.792
Hyperlipidemia no. (%)	21 (95.5)	41 (78.8)	3.139	0.587
Coronary heart disease no. (%)	0 (0)	2 (3.8)	0.870	0.351
BMI of 30 or more no. (%)	3 (13.6)	3 (5.8)	1.284	0.257

Table 1 shows that 22 patients have no carotid plaque and 52 patients belonged to the carotid plaque group; there were no significant differences found between the groups in years, gender, diabetes, hypertension, hyperlipidemia, coronary heart disease, and BMI.

**Figure 4:**
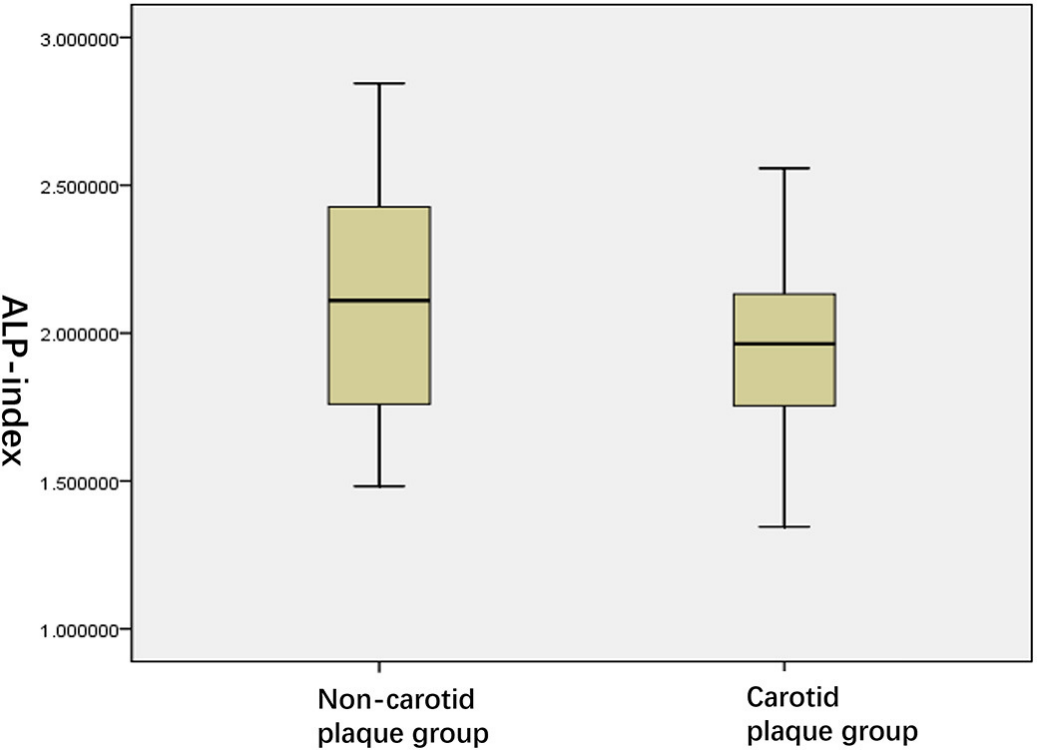
ALPS-index in two groups.

**Figure 5:**
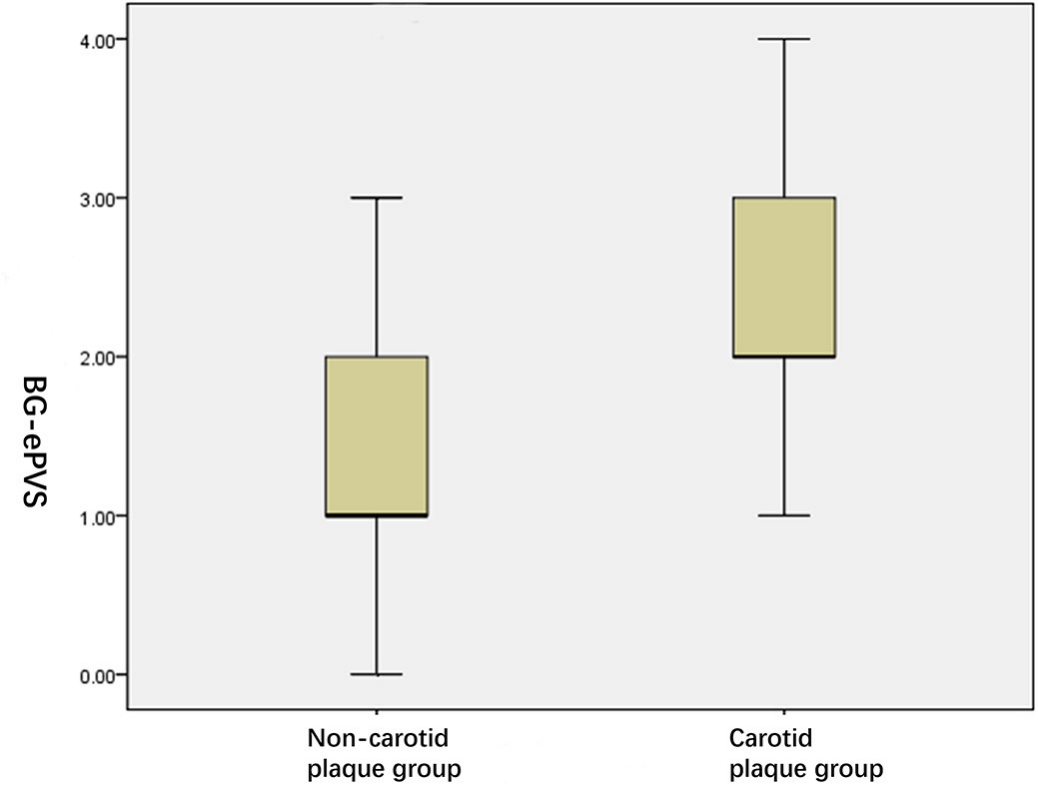
High grade BG-ePVS in two groups.

As shown in Table 2, between the two groups, ALPS-index values in carotid plaque group were significantly lower than that of the non-carotid plaque group (*P* = 0.034, Figure 4); conversely, IMT in carotid plaque group were significantly higher than that of non-carotid plaque group (*P* = 0.034, Figure 4). Moreover, compared with non-carotid plaque group, there were more ePVS high grade and score in BG (*P* = 0.000, Figure 5).

**Table 2:** Imaging index and blood examination.

	**Non-carotid plaque group (*n* = 22)**	**Carotid plaque group (*n* = 52)**	**χ^**2**^/*t* value/ *z* value**	***P*-value**
ALPS-index	2.12 ± 0.39	1.95 ± 0.28	2.167	0.034^*^
BG-ePVS (high grade) no. (%)	9 (40.9)	44 (84.6)	14.529	0.000^*^
CSO-ePVS (high grade) no. (%)	6 (27.3)	7 (13.5)	2.036	0.154
BG-ePVS score	25.68	42.50	−3.127	0.001^*^
CSO-ePVS score	41.45	35.83	−1.085	0.278
Mean arterial pressure	102.86 ± 10.80	104.81 ± 11.64	−0.670	0.505
Pulse pressure	50.32 ± 9.54	50.94 ± 15.00	−0.18	0.858
IMT	0.72 ± 0.08	0.80 ± 0.10	−3.415	0.001^*^
MMSE	29.05 ± 1.00	28.58 ± 1.16	1.651	0.103
FPG	6.32 ± 0.26	6.25 ± 2.22	0.120	0.905
TG	2.31 ± 1.80	1.95 ± 0.81	1.204	0.232
LDL-c	3.34 ± 1.03	3.42 ± 0.88	−0.373	0.711
IL-6	72.27 ± 37.55	69.24 ± 26.88	0.392	0.696
hsCRP	1.54 ± 1.14	1.18 ± 1.26	−1.009	0.316

* *represents that the difference is statistically significant*.

Table 3 demonstrates that ALPS-index was negatively correlated with IMT (*r* = −0.262, *P* = 0.024) and BG-ePVS score (*r* = −0.242, *P* = 0.038) whereas no significant correlation was found between ALPS-index and CSO-ePVS score (*r* = −1.085, *P* = 0.278). Meanwhile, ALPS-index showed no significant correlations with FPG, IL-6, hsCRP, cognitive function score (MMSE), pulse pressure, etc. ALPS-index has no significant correlations with gentle either, as shown in Table 4.

**Table 3:** Relationship between ALPS-index and other variables.

	**Variable**	***R***	***P*-value**
ALPS-index	BG-ePVS score	−0.242^*^	0.038^*^
	CSO-ePVS score	−0.006	0.960
	Systolic pressure	0.059	0.619
	Diastolic blood pressure	−0.003	0.983
	Pulse pressure	−0.078	0.509
	FPG	−0.071	0.549
	IMT	−0.262^*^	0.024^*^
	IL-6	0.003	0.980
	hsCRP	−0.054	0.645
	Cholesterol	0.091	0.443
	MMSE	0.041	0.730
	Age	0.084	0.477

* *represents that the difference is statistically significant*.

**Table 4:** ALPS-index in different sex groups.

	**Male (*n* = 32)**	**Female (*n* = 42)**	***t*-value**	***P*-value**
ALPS-index	1.97 ± 0.35	2.02 ± 0.30	−0.652	0.516

Table 5 shows that the effect of IMT and BG-ePVS score on ALPS-index was statistically significant (*P* < 0.05).

**Table 5:** Multiple linear regression analysis of ALPS-index and other variables.

**Variable**	**Coefficients**	***t*-value**	***P*-value**
Age	0.040	0.345	0.731
MMSE	0.181	1.579	0.119
IMT	−0.249	−2.238	0.028^*^
BG-ePVS score	0.256	2.257	0.027^*^

* *represents that the difference is statistically significant*.

## Discussion

There are growing interests in the fluid loop circuit in the glymphatic system and driving mechanisms for this interchange. Transmission in the PVS is believed to be an advection and it is generated by the cardio-cerebrovascular pulsatility in each cardiac cycle on the brain parenchyma (10, 11, 31, 32).

Recently, there are several studies that have made use of the ALPS-index to evaluate the function of cerebral glymphatic system (16, 18–20). We calculated the ALPS-index in order to clarify the activity of the glymphatic system in asymptomatic patients with carotid plaque and controls. There are several important findings in this study. First of all, we found that there were significant differences in ALPS-index between carotid plaque group and non-carotid plaque group using the means of the groups in an independent-sample *t*-test (Table 2). ALPS-index in the carotid plaque group is lower than that in the non-carotid plaque group. This finding is evident of the dysfunction of glymphatic system in the early and middle stages of CAS. This result is similar to the results of the research by Yang et al. on T2DM (20). Asymptomatic patients with carotid plaque might have a common feature of cortical atherothrombotic embolism and lacunar stroke; the coexisting macrovascular and microvascular damage can be induced by different degrees of carotid plaque, increasing the risk of developing small vessel disease with the enlargement of the PVS (11, 33). The results were consistent with those of previous studies, specifically those that showed that carotid atherosclerosis is associated with brain changes (34, 35). Our research supports the hypothesis that CAS is related to changes in the brain injury and the cardiovascular and cerebrovascular diseases can affect the brain structure and brain function (25, 36), which might be achieved by affecting the function of the glymphatic system. It is interesting to note that comparing asymptomatic patients with carotid plaque and non-carotid plaque, the correlation analysis showed that the ALPS-index was not a significant sign correlated with sex, age, MBP, FBS, TG, LDL-c, cognitive function score (MMSE), pulse pressure, history of hypertension, diabetes, and hypercholesterolemia.

Our study suggested that CAS is directly related to the glymphatic system of the brain (37). Previous studies have speculated that the expansion of the PVS is evident of intracranial imaging lesions caused by the dysfunction of the glymphatic system (38). However, so far, studies using the DTI-ALPS method assessing the function of the glymphatic system lack the analysis of ePVS of the study subjects. Our study examined the relationship between MRI-visible ePVS and DTI-ALPS. Further analysis showed that there are more high-grade ePVS in the BG area compared to the carotid plaque group with the non-carotid plaque group; the ALPS-index showed significantly negative correlation with the BG-ePVS score while there was no significant difference in the CSO-ePVS score. This result further confirms the hypothesis that CAS affects the function of the glymphatic system and causes PVS in BG-ePVS. We know that the expansion of PVS in the BG is related to blood pressure variability (39) and is related to deep perforating atherosclerosis (40). In our study, there was no significant difference in the variability of blood pressure or pulse pressure between the two groups. The reasons may be as follows: First, there may be other mechanisms involved in carotid plaque, deep perforating artery atherosclerosis, DTI-ALPS changes and glymphatic system dysfunction, BG-ePVS. Second, due to the deviation of the study, for example, the sample size is too small. The sample will be expanded for further research in the future, but it cannot be ignored that it is very likely that CAS itself can mediate the decrease in the clearance of the glymphatic system.

Clinical research data have proved that ePVS is a risk factor for cognitive impairment and dementia. The ePVS is an important risk factor for cognitive decline in Parkinson's disease and Alzheimer's disease, as well as vascular cognitive impairment and vascular dementia (21, 41–43). However, we found no significant difference between MMSE and BG-ePVS score and CSO-ePVS score. The correlation analysis also showed that the ALPS-index had no significant signs correlated with the MMSE score. The glymphatic system is a covert discovered waste drainage system in the brain parenchyma that involves the movement of the CSF along the PVS. This study further confirms that cognitive dysfunction is the result of abnormalities in the glymphatic system rather than the cause. In the early stages of atherosclerosis, MMSE score is not a sensitive indicator of vascular cognitive decline. Previous research findings of our studies (44), using ASL technique to evaluate mean CBF of the whole brain, found normal cognitive functions and CBF in two groups. In other words, the phenomenon that CAS affects the function of the glymphatic system and causes the perivascular space in the basal ganglia to expand might not be caused by decreased cerebral perfusion.

We investigated the associations between inflammation biomarkers and the ALPS-index changes in asymptomatic patients with carotid plaque and control group to further evaluate the pathophysiological mechanism of the glymphatic system in individuals with cerebral arteriosclerosis before stroke and dementia, such as serum IL-6 and hsCRP. But the results showed that the ALPS-index was not a significant sign correlated with inflammation biomarkers mentioned above, which may be attributed to the insufficiency of sample size or the possibility that systemic inflammation may not affect the CSF along the ePVS.

In this pilot study, limitations included small sample size in our single center; further multiple center studies with larger samples are needed to assist in improving the usefulness of the DTI-ALPS parameter and whether it may be a biomarker of neuro-fluid dynamics in the glymphatic system. Due to the limited sample size, we failed to further analyze other different types of CAS, such as carotid vulnerable/stable plaque and carotid stenosis. Second, the ROIs were placed manually, and the ePVS score were counted manually, which might be a subjective factor of our measurement. Third, the method of the ALPS-index is theoretically deductive. In future, even if the components of carotid plaque are related to the function of the glymphatic system, it is necessary to combine the high-resolution MRI of carotid and high-level DTI parameter analysis, which will be a very meaningful task.

## Conclusion

In summary, the present study provides elementary evidence that carotid plaque may reduce DTI-ALPS, damage the function of the glymphatic system, and promote the expansion of the PVs in the BG. This study revealed novel interesting evidence indicating that alterations in ALPS-index are associated with early high-risk cerebrovascular diseases, helping to find out the actual damage of carotid plaque in patients with early dysfunction of intracranial neurovascular unit, looking for the early pathophysiological changes from the micro level.

## Data Availability Statement

The raw data supporting the conclusions of this article will be made available by the authors, without undue reservation.

## Ethics Statement

The studies involving human participants were reviewed and approved by the Ethics Committee of Xiangya Hospital of Central South University. The patients/participants provided their written informed consent to participate in this study.

## Author Contributions

HL and SY: data analysis, statistical analysis, and manuscript preparation. WH and XL: data acquisition and data analysis. SS and SW: data acquisition. YW, XZ, TT, JX, and YL: data acquisition and definition of intellectual content. QH: study concepts, study design, and definition of intellectual content, guarantor of integrity of the entire study. All authors contributed to the article and approved the submitted version.

## Funding

The study was funded by the National Natural Science Foundation of China (Grant No. 81400978) and the Youth Program of National Natural Science Foundation of China (No. 81801365).

## Conflict of Interest

The authors declare that the research was conducted in the absence of any commercial or financial relationships that could be construed as a potential conflict of interest.

## Publisher's Note

All claims expressed in this article are solely those of the authors and do not necessarily represent those of their affiliated organizations, or those of the publisher, the editors and the reviewers. Any product that may be evaluated in this article, or claim that may be made by its manufacturer, is not guaranteed or endorsed by the publisher.

## References

[1] Al Hazzouri AZ Vittinghoff E Sidney S Reis JP Jacobs Jr DR Yaffe K. Intima-media thickness and cognitive function in stroke-free middle-aged adults: findings from the coronary artery risk development in young adults study. Stroke. (2015) 46:2190–6. 10.1161/STROKEAHA.115.008994 26106116 PMC4519386

[2] Meschia JF Bushnell C Boden-Albala B Braun LT Bravata DM Chaturvedi S. Guidelines for the primary prevention of stroke: a statement for healthcare professionals from the American Heart Association/American Stroke Association. Stroke. (2014) 45:3754–832. 10.1161/STR.0000000000000046 25355838 PMC5020564

[3] Zhong WJ Cruickshanks KJ Schubert CR Acher CW Carlsson CM Klein BEK. Carotid atherosclerosis and 10-year changes in cognitive function. Atherosclerosis. (2012) 224:506–10. 10.1016/j.atherosclerosis.2012.07.024 22854188 PMC3459157

[4] Suemoto CK Santos IS Bittencourt MS Pereira AC Goulart AC Rundek T. Subclinical carotid artery atherosclerosis and performance on cognitive tests in middle-aged adults: baseline results from the ELSA-Brasil. Atherosclerosis. (2015) 243:510–5. 10.1016/j.atherosclerosis.2015.10.008 26520907

[5] Barisano G Sheikh-Bahaei N Law M Toga AW Sepehrband F. Body mass index, time of day and genetics affect perivascular spaces in the white matter. J Cereb Blood Flow Metab. (2021) 41:1563–78. 10.1177/0271678X20972856 33183133 PMC8221772

[6] Wardlaw JM Benveniste H Nedergaard M Zlokovic BV Mestre H Lee H. Perivascular spaces in the brain: anatomy, physiology and pathology. Nat Rev Neurol. (2020) 16:137–53. 10.1038/s41582-020-0312-z 32094487

[7] Hilal S Tan CS Adams HHH Habes M Mok V Venketasubramanian N. Enlarged perivascular spaces and cognition: a meta-analysis of 5 population-based studies. Neurology. (2018) 91:E832–42. 10.1212/WNL.0000000000006079 30068634 PMC6133622

[8] Xia YW Shen Y Wang Y Yang LM Wang YQ Li Y. White matter hyperintensities associated with progression of cerebral small vessel disease: a 7-year Chinese urban community study. Aging. (2020) 12:8506–22. 10.18632/aging.103154 32388497 PMC7244059

[9] Li XD Shen MX Jin Y Jia SH Zhou Z Han ZL. The effect of cerebral small vessel disease on the subtypes of mild cognitive impairment. Front Psychiatry. (2021) 12:685965. 10.3389/fpsyt.2021.685965 34335331 PMC8322581

[10] Iliff JJ Wang MH Liao YH Plogg BA Peng WG Gundersen GA. A paravascular pathway facilitates CSF flow through the brain parenchyma and the clearance of interstitial solutes, including amyloid beta. Sci. Transl. Med. (2012) 4:147ra11. 10.1126/scitranslmed.3003748 22896675 PMC3551275

[11] Mestre H Tithof J Du T Song W Peng WG Sweeney AM. Flow of cerebrospinal fluid is driven by arterial pulsations and is reduced in hypertension. Nat Commun. (2018) 9:4878. 10.1038/s41467-018-07318-3 30451853 PMC6242982

[12] Nedergaard M Goldman SA. Glymphatic failure as a final common pathway to dementia. Science. (2020) 370:50–6. 10.1126/science.abb8739 33004510 PMC8186542

[13] Mestre H Mori Y Nedergaard M. The brain's glymphatic system: current controversies. Trends Neurosci. (2020) 43:458–66. 10.1016/j.tins.2020.04.003 32423764 PMC7331945

[14] Plog BA Nedergaard M. The glymphatic system in central nervous system health and disease: past, present, and future. Annu Rev Pathol. (2018) 13:379–94. 10.1146/annurev-pathol-051217-111018 29195051 PMC5803388

[15] Rasmussen MK Mestre H Nedergaard M. The glymphatic pathway in neurological disorders. Lancet Neurol. (2018) 17:1016–24. 10.1016/S1474-4422(18)30318-1 30353860 PMC6261373

[16] Taoka T Masutani Y Kawai H Nakane T Matsuoka K Yasuno F. Evaluation of glymphatic system activity with the diffusion MR technique: diffusion tensor image analysis along the perivascular space (DTI-ALPS) in Alzheimer's disease cases. Jpn JRadiol. (2017) 35:172–8. 10.1007/s11604-017-0617-z 28197821

[17] Zhou W Shen B Shen WQ Chen H Zheng YF Fei JJ. Dysfunction of the glymphatic system might be related to iron deposition in the normal aging brain. Front Aging Neurosci. (2020) 12:559603. 10.3389/fnagi.2020.559603 33408625 PMC7779624

[18] Chen HL Chen PC Lu CH Tsai NW Yu CC Chou KH. Associations among cognitive functions, plasma DNA, and diffusion tensor image along the perivascular space (DTI-ALPS) in patients with Parkinson's disease. Oxid Med Cell Longev. (2021) 2021:4034509. 10.1155/2021/4034509 33680283 PMC7904342

[19] Yokota H Vijayasarathi A Cekic M Hirata Y Linetsky M Ho M. Diagnostic performance of glymphatic system evaluation using diffusion tensor imaging in idiopathic normal pressure hydrocephalus and mimickers. Curr Gerontol Geriatr Res. (2019) 2019:5675014. 10.1155/2019/5675014 31320896 PMC6609364

[20] Yang GW Deng N Liu Y Gu YJ Yao X. Evaluation of glymphatic system using diffusion MR technique in T2DM cases. Front Hum Neurosci. (2020) 14:300. 10.3389/fnhum.2020.00300 32922272 PMC7456821

[21] Steward CE Venkatraman VK Lui E Malpas CB Ellis KA Cyarto EV. Assessment of the DTI-ALPS parameter along the perivascular space in older adults at risk of dementia. J Neuroimaging. (2021) 31:569–78. 10.1111/jon.12837 33556226

[22] Vizcarra JA Lang AE Sethi KD Espay AJ. Vascular parkinsonism: deconstructing a syndrome. Mov Disord. (2015) 30:886–94. 10.1002/mds.26263 25997420 PMC4478160

[23] Folstein MF Folstein SE McHugh PR. Mini-mental state. A practical method for grading the cognitive state of patients for the clinician. J Psychiatr Res. (1975) 12:189–98. 10.1016/0022-3956(75)90026-6 1202204

[24] Pignoli P Tremoli E Poli A Oreste P Paoletti R. Intimal plus medial thickness of the arterial wall: a direct measurement with ultrasound imaging. Circulation. (1986) 74:1399–406. 10.1161/01.CIR.74.6.1399 3536154

[25] Liu LH Huang Q Yang S Wen YB He W Liu H. Micro-structural white matter abnormalities and cognitive impairment in asymptomatic carotid plaque patients: a DTI study using TBSS analysis. Clin Neurol Neurosurg. (2020) 197:106096. 10.1016/j.clineuro.2020.106096 32717561

[26] Potter GM Doubal FN Jackson CA Chappell FM Sudlow CL Dennis MS. Enlarged perivascular spaces and cerebral small vessel disease. Int J Stroke. (2015) 10:376–81. 10.1111/ijs.12054 23692610 PMC4463944

[27] Potter GM Chappell FM Morris Z Wardlaw JM. Cerebral perivascular spaces visible on magnetic resonance imaging: development of a qualitative rating scale and its observer reliability. Cerebrovasc Dis. (2015) 39:224–31. 10.1159/000375153 25823458 PMC4386144

[28] Taoka T Ito R Nakamichi R Kamagata K Sakai M Kawai H. Reproducibility of diffusion tensor image analysis along the perivascular space (DTI-ALPS) for evaluating interstitial fluid diffusivity and glymphatic function: CHanges in Alps index on Multiple conditiON acquIsition eXperiment (CHAMONIX) study. Jpn JRadiol. (2021). 10.1007/s11604-021-01187-5 PMC8803717 34390452

[29] Bae YJ Choi BS Kim JM Choi JH Cho SJ Kim JH. Altered glymphatic system in idiopathic normal pressure hydrocephalus. Parkinsonism Relat Disord. (2021) 82:56–60. 10.1016/j.parkreldis.2020.11.009 33248394

[30] Masutani Y Aoki S Abe O Hayashi N Otomo K. MR diffusion tensor imaging: recent advance and new techniques for diffusion tensor visualization. Eur J Radiol. (2003) 46:53–66. 10.1016/S0720-048X(02)00328-5 12648802

[31] Iliff JJ Wang MH Zeppenfeld DM Venkataraman A Plog BA Liao YH. Cerebral arterial pulsation drives paravascular CSF-interstitial fluid exchange in the murine brain. J Neurosci. (2013) 33:18190–9. 10.1523/JNEUROSCI.1592-13.2013 24227727 PMC3866416

[32] Rennels ML Gregory TF Blaumanis OR Fujimoto K Grady PA. Evidence for a 'paravascular' fluid circulation in the mammalian central nervous system, provided by the rapid distribution of tracer protein throughout the brain from the subarachnoid space. Brain Res. (1985) 326:47–63. 10.1016/0006-8993(85)91383-6 3971148

[33] Zhai FF Yang M Wei Y Wang M Gui Y Han F. Carotid atherosclerosis, dilation, and stiffness relate to cerebral small vessel disease. Neurology. (2020) 94:E1811–9. 10.1212/WNL.0000000000009319 32241954

[34] Wang H Nie ZY Liu M Li RR Huang LH Lu Z. Clinical characteristics of perivascular space and brain CT perfusion in stroke-free patients with intracranial and extracranial atherosclerosis of different extents. Ann Transl Med. (2020) 8:215. 10.21037/atm.2020.01.35 32309362 PMC7154435

[35] Tuo J Huang Q He W Yang S Liu LH Liu XJ. Disrupted topological organization of functional networks in asymptomatic carotid plaque without significant carotid stenosis: a resting-state fMRI study. Front Hum Neurosci. (2021) 15:685763. 10.3389/fnhum.2021.685763 34421560 PMC8375554

[36] Berman SE Wang X Mitchell CC Kundu B Jackson DC Wilbrand SM. The relationship between carotid artery plaque stability and white matter ischemic injury. Neuroimage Clin. (2015) 9:216–22. 10.1016/j.nicl.2015.08.011 26448914 PMC4572385

[37] Wang JQ Huang R Tian S Lin HY Guo D An K. Elevated plasma level of D-dimer predicts the high risk of early cognitive impairment in type 2 diabetic patients as carotid artery plaques become vulnerable or get aggravated. Curr Alzheimer Res. (2019) 16:396–404. 10.2174/1567205016666190321164741 30919777

[38] Williamson W Lewandowski AJ Forkert ND Griffanti L Okell TW Betts J. Association of cardiovascular risk factors with MRI indices of cerebrovascular structure and function and white matter hyperintensities in young adults. JAMA. (2018) 320:665–73. 10.1001/jama.2018.11498 30140877 PMC6142949

[39] Mestre H Kostrikov S Mehta RI Nedergaard M. Perivascular spaces, glymphatic dysfunction, and small vessel disease. Clin Sci. (2017) 131:2257–74. 10.1042/CS20160381 28798076 PMC5567781

[40] Yang SN Yuan JL Zhang XY Fan HM Li Y Yin JM. Higher ambulatory systolic blood pressure independently associated with enlarged perivascular spaces in basal ganglia. Neurol Res. (2017) 39:787–94. 10.1080/01616412.2017.1324552 28475469

[41] Paradise M Crawford JD Lam BP Wen W Kochan NA Makkar S. Association of dilated perivascular spaces with cognitive decline and incident dementia. Neurology. (2021) 96:E1501–11. 10.1212/WNL.0000000000011537 33504642 PMC8032377

[42] Fang Y Gu LY Tian J Dai SB Chen Y Zheng R. MRI-visible perivascular spaces are associated with cerebrospinal fluid biomarkers in Parkinson's disease. Aging. (2020) 12:25805–18. 10.18632/aging.104200 33234732 PMC7803484

[43] Schneider ALC Rawlings AM Sharrett AR Alonso A Mosley TH Hoogeveen RC. High-sensitivity cardiac troponin T and cognitive function and dementia risk: the atherosclerosis risk in communities study. Eur Heart J. (2014) 35:1817–24. 10.1093/eurheartj/ehu124 24685712 PMC4097965

[44] Huang Q Liu YH Liao WH Yang S Shen L Tang T. Disruption of regional brain activity and functional connectivity in patients with asymptomatic vulnerable carotid plaque. Neurosci Lett. (2020) 716:134634. 10.1016/j.neulet.2019.134634 31751668

